# Cytogenetic Analysis and Molecular Marker Development for a New Wheat–*Thinopyrum ponticum* 1J^s^ (1D) Disomic Substitution Line With Resistance to Stripe Rust and Powdery Mildew

**DOI:** 10.3389/fpls.2020.01282

**Published:** 2020-08-21

**Authors:** Yanzhen Wang, Qiang Cao, Junjie Zhang, Siwen Wang, Chunhuan Chen, Changyou Wang, Hong Zhang, Yajuan Wang, Wanquan Ji

**Affiliations:** ^1^State Key Laboratory of Crop Stress Biology for Arid Areas and College of Agronomy, Northwest A&F University, Yangling, China; ^2^Shaanxi Research Station of Crop Gene Resources and Germplasm Enhancement, Ministry of Agriculture, Yangling, China

**Keywords:** *Thinopyrum ponticum*, wide hybridization, wheat 15K array, *Pst*, *Bgt* SLAF-seq

## Abstract

*Thinopyrum ponticum* (2*n* = 10*x* = 70), a member of the tertiary gene pool of wheat (*Triticum aestivum* L.), harbors many biotic and abiotic stress resistance genes. CH10A5, a novel disomic substitution line from a cross of *T. aestivum* cv. 7182 and *Th. ponticum*, was characterized by cytogenetic identification, *in situ* hybridization, molecular marker analysis, and morphological investigation of agronomic traits and disease resistance. Cytological observations showed that CH10A5 contained 42 chromosomes and formed 21 bivalents at meiotic metaphase I. Genome *in situ* hybridization (GISH) analysis indicated that two of its chromosomes came from the J^s^ genome of *Th. ponticum*, and wheat 15K array mapping and fluorescence *in situ* hybridization (FISH) revealed that chromosome 1D was absent from CH10A5. Polymorphic analysis of molecular markers indicated that the pair of alien chromosomes belonged to homoeologous group one, designated as 1J^s^. Thus, CH10A5 was a wheat–*Th. ponticum* 1J^s^ (1D) disomic substitution line. Field disease resistance trials demonstrated that the introduced *Th. ponticum* chromosome 1J^s^ was probably responsible for resistance to both stripe rust and powdery mildew at the adult stage. Based on specific-locus amplified fragment sequencing (SLAF-seq), 507 STS molecular markers were developed to distinguish chromosome 1J^s^ genetic material from that of wheat. Of these, 49 STS markers could be used to specifically identify the genetic material of *Th. ponticum*. CH10A5 will increase the resistance gene diversity of wheat breeding materials, and the markers developed here will permit further tracing of heterosomal chromosome fragments in the future.

## Introduction

Epidemics of stripe rust, caused by *Puccinia striiformis* f. sp. *tritici* (*Pst*), occur frequently in areas with cool conditions during the wheat growing season. Powdery mildew is caused by *Blumeria graminis* (DC.) E.O.f. sp. *tritici* (*Bgt*) (An et al., 2019). These two major diseases can dramatically reduce crop yield during prevalent years (Bokore et al., 2017). Numerous powdery mildew and stripe rust resistance genes have been identified in wheat and its relatives, and these genes have played a fundamental role in wheat breeding (Liu et al., 2019). Nevertheless, most resistance genes are quickly overcome due to rapid virulence changes in pathogen populations (Zhan et al., 2015). Therefore, it is extremely important to identify new resistance genes, especially broad-spectrum resistance genes, from wheat-related species.

*Thinopyrum ponticum* (2*n* = 10*x* = 70) often produces large amount of biomass and is tolerant of biotic and abiotic stresses such as drought and salt stress. It is also highly resistant to wheat leaf rust, stem rust, stripe rust, powdery mildew, barley yellow dwarf virus (BYDV), and *Fusarium* head blight (FHB) (Ceoloni et al., 2014; Kroupin et al., 2019), and recently, *Fhb7* was successfully cloned by Wang et al. (2020a) from *Th. elongatum*. Many stripe rust and powdery mildew resistance genes are derived from *Th. ponticum* and have been introduced to common wheat by additions, substitutions, translocations, and introgression of chromosomes. Despite the development of numerous wheat*–Th. ponticum* chromosome lines, addition or substitution lines of homoeologous group one are rarely reported (Mo et al., 2017; Zhu et al., 2017; Mago et al., 2019; Wang et al., 2019), and only a few stripe rust and powdery mildew resistance genes have been introgressed into cultivated wheat varieties (Li et al., 2003; Zhan et al., 2014; Hou et al., 2016). The resistance genes of *Th. ponticum* have not been fully characterized. Therefore, the creation and identification of new wheat*–Th. ponticum* derivative lines carrying new resistance genes are important for the expansion of wheat genetic resources (Ceoloni et al., 2017). During wheat distant hybridization, rapid tracing and accurate identification of alien fragments directly affect the development of new wheat varieties and the process of breeding (Ceoloni et al., 2014).

Molecular marker analysis is the most convenient means for identifying exogenous chromosomes or chromosome segments. Wild triticeae grasses have revealed the presence of multiple homoeologous gene copies and repetitive DNA compared with common wheat, which were helpful to molecular marker development and also chromosome identification (Li et al., 2016). Nonetheless, existing markers do not meet the demand for *Th. ponticum* chromatin detection (Liu et al., 2018b), and it is therefore particularly important to develop chromosome-specific markers for the detection of *Th. ponticum* genetic materials. At present, next generation sequencing is the most important technology for molecular marker development and includes such techniques as RNA-seq (Okada et al., 2018), whole genome sequencing (Tiwari et al., 2015), genotyping-by-sequencing (Elshire et al., 2011), and specific-locus amplified fragment sequencing (SLAF-seq) (Sun et al., 2013). SLAF-seq refers to a sequencing strategy in which restriction enzymesare used to fragment genomic DNA and high-throughput sequencing is performed on a number of specific fragments in order to fully represent the genomic information of the target species. SLAF-seq plays a fundamental role in molecular breeding, gene location, and germplasm resource identification. It has been used to develop specific markers in wheat-related species with high efficiencies, up to 66% (Chen et al., 2013). SLAF-seq can be employed to obtain sufficient markers for the construction of a high-density genetic map and permit the exploitation of useful genes for wheat breeding applications.

In this study, a novel wheat*–Th. ponticum* 1J^s^ (1D) disomic substitution line with resistance to stripe rust and powdery mildew was developed from the F_6_ progeny of a wheat*–Th. ponticum* hybrid and named CH10A5. We used cytogenetic methods to assess the stability of CH10A5 through observations of chromosome pairing. Its chromosomal composition was determined using fluorescence *in situ* hybridization (FISH), simple sequence repeats (SSR) markers, expressed sequence tag sequence-tagged sites (EST-STS) markers, PCR-based landmark unique gene (PLUG) markers, and a single-nucleotide polymorphism (SNP) array. Its agronomic and disease resistance characteristics were also assessed. This is the first report of stripe rust and powdery mildew resistance on chromosome 1J^s^. Specific molecular markers of 1J^s^ were developed using SLAF-seq and will play a very important role in reducing the length of the breeding cycle and improving selection efficiency.

## Materials and Methods

### Plant Materials and Isolation Scheme of the CH10A5 Line

The wheat-related species used in this study included *Thinopyrum ponticum* (2*n* = 10*x* = 70, E^e^E^e^E^b^E^b^E^x^E^x^StStStSt/JJJJJJJ^s^J^s^J^s^J^s^), *Th. bessarabicum* (2 = 2*x* = 14, E^b^E^b^/JJ), *Th. elongatum* (2*n* = 2*x* = 14, E^e^E^e^/EE), tetraploid *Pseudoroegeria spicata* (2*n* = 4*x* = 28, StStStSt), *Th. intermedium* (2*n* = 6*x* = 42, E^e^E^e^E^b^E^b^StSt/JJJsJsStSt)*, Leymus mollis* (Trin.) Pilger, (2*n* = 4*x* = 28, NsNsXmXm), *Psathyrostachys huashanica* (2*n* = 2*x* = 14, NsNs)*, Secale cereale* L. (2*n* = 2*x* = 14, RR*), Aegilops geniculata* Roth. (2*n* = 4*x* = 28, UUMM), *Triticum urartu* Thum. (2*n* = 2*x* = 14, AA), and *Ae. tauschii* Coss. (2*n* = 2*x* = 14, DD).

The common wheat cultivar 7182 (2*n* = 6*x* = 42, AABBDD) was used as the male parent. Shanyou225 (SY225) was used as the susceptible control for *Bgt*, and Huixianhong (HXH) and Mingxian169 (MX169) were used as the susceptible controls for *Pst*. Chinese Spring (CS) was used as a blocker in GISH analysis, and nulli-tetrasomic materials (CSN1AT1B, CSN1BT1D, and CSN1DT1B) were used in molecular marker detection.

The isolation scheme of the CH10A5 line began with hybridization of the female parent, *Th. ponticum*, with 7182 in 2009. The resulting F_1_ progeny were backcrossed with 7182, then 18 lines with good morphologically characteristics were selected in the year of 2011, such as long spikes, strong resistance to stripe rust and powdery mildew, and so on. The chromosomes of root tip cells and their pairing in pollen mother cells (PMCs) was observed in each generation. After 6 years of strict self-crossing, a total of 375 lines were identified in BC_I_F_6_ generation, in which 136 lines had 42 chromosomes in root tip cells, accounting for 36%. CH10A5 was finally selected in BC_I_F_6_ generation (June 2016). All plant materials were preserved in our laboratory at the College of Agronomy, Northwest A&F University, China.

### Cytological Stability Assessment

Root tip cells and PMCs of CH10A5 from the same plant were observed for three growing seasons (2016–2019) in a total of 34 plants. Root tip processing procedures were performed as described in Zheng et al. (2006), using a mixture of cellulase (R-10, Yakult Japan) and pectinase (Y-23, Yakult Japan) for cell enzymatic hydrolysis (Zhu et al., 2017). Young spikes (Zadoks stage 45) were sampled, and anthers at meiosis metaphase I (MI) were fixed in Carnoy fixative (a 6:3:1 ethanol-chloroform-acetic acid mixture) for at least 2 days and stained with a 1% (w/v) aceto-carmine solution prior to *in situ* hybridization of the PMCs as described in Yang et al. (2014). Chromosomes were fixed for 60s at an ultraviolet intensity of 125,000μJ/cm^2^ by UV irradiation (Spectrolinker™ XL-1500, USA). Cells were examined with an Olympus BX43 microscope (Japan) equipped with a Photometrics SenSys CCD camera.

### *In Situ* Hybridization of CH10A5

The total genomic DNA was extracted from fresh leaves using a modified CTAB method (Allen et al., 2006) with one additional purification step to obtain high-quality DNA. The total genomic DNA of *Th. ponticum* was labeled with Red-5-dUTP (Invitrogen) by nick translation (Nick Translation Mix, Roche). Genomic DNA of CS was fixed in boiling water for 5 min and used as a blocker at a ratio of 1:300. The GISH procedure was performed as described in Fu et al. (2012). For multicolor GISH (mc-GISH) analysis, *Th. bessarabicum* DNA was labeled with Alexa Fluor 488-5-dUTP (Invitrogen), and *Ps. spicata* DNA was labeled with Texas Red-5-dUTP (Invitrogen) (Wang et al., 2019).

The oligonucleotide probes used to identify wheat and *Th. ponticum* chromosomes by non-denaturing FISH (ND-FISH) analysis included Oligo-pSc119.2 (6-FAM-5′), Oligo-pTa535 (Tamra-5′) (Tang et al., 2014), Oligo-44 (6-FAM-5′), and Oligo-D (6-FAM-5′) (Tang et al., 2018), all of which were synthesized by Shanghai Invitrogen Biotechnology Co. Ltd. (Shanghai, China). Chromosomes were counterstained with 4,6-diamidino-2-phenylindole (DAPI). Fluorescent signals were scanned and photographed with an Olympus BX53 microscope equipped with a Photometrics SenSys CCD DP80 camera (Japan) (Wang et al., 2016).

### Homoeologous Group Identification

#### Wheat SNP Array Analysis

Genomic DNA of CH10A5 and its parents was hybridized to wheat 15K SNP genotyping arrays by China Golden Marker Biotechnology Company (Beijing, China). There were 13,947 microchip probes per chip, including a large number of diploid markers. Based on IWGSC-RefSeq-v1.0 (https://wheat-urgi.versailles.inra.fr/Seq Repository/), a total of 13,199 markers had explicit physical location information, evenly covering the entire wheat genome. Data processing and analysis of the 15K array were performed to analyze polymorphic markers using SigmaPlot V12.5 (SYSTAT software, Inc. USA) as described in Li et al. (2019).

#### Molecular Markers and Nulli-Tetrasomic Analysis

SSR markers, EST-STS markers (http://wheat.pw.usda.gov/SNP/new/pcr_primers.shtml) and PLUG markers for wheat homoeologous groups one to seven were synthesized by AuGCT DNA-SYN Biotechnology Co., Ltd of Beijing (Ishikawa et al., 2009). A total of 674 markers were used to identify the homoeologous group of the exogenous chromosomes carried by CH10A5. These included 467 SSR markers, 72 EST markers, and 135 PLUG markers **(****Supplementary Table 1**). The polymerase chain reaction (PCR) amplification system contained: 10×PCR Buffer (Mg^2+^) 1.0 μl, 0.02 mM/μl dNTPs, 0.05 μM/μl primers, 0.05 U/μl Taq DNA polymerase (Takara), 10 ng/μl DNA, and ddH_2_O up to 10.0 µl. The PCR products were then digested with *Taq Ⅰ* (Takara) and *Hae Ⅲ* (Takara) (Zhu et al., 2017). The reagent for the PCR reaction was purchased from Takara Biomedical Technology (Beijing) Co., Ltd.

### Disease Resistance Assessment

Stripe rust races CYR23, CYR29, CYR31, CYR 32, CYR33, and CYR 34 were kindly donated by the College of Plant Protection, Northwest A&F University, China. Disease resistance was investigated three times each year, and the most severe reaction type observed in a given year was considered to be the final disease resistance result.

Seedling stripe rust resistance trials were performed in an incubator. Each of five *Pst* races (CYR23, CYR29, CYR31, CYR32, and CYR34) was mixed with talcum powder at a 1:30 ratio using the shaking powder method. When seedlings had grown to the two-leaf stage, 10 plants in a pot were inoculated with one of the powder mixtures, and each treatment was replicated three times. The temperature in the incubator was 17°C during the day and 11°C at night. Day length was 16 h (6000 lux), and the relative humidity was 70%. When the susceptible control had become fully affected, we began to assess disease resistance every 4 days until 21 days after inoculation.

Adult stripe rust resistance trials were performed under field conditions over three growing seasons (2016–2019) using mixtures of *Pst* races from Shaanxi Province, including CYR31, CYR32, CYR33, and CYR34 (Zhan et al., 2015). The races were inoculated by using the shaking powder method at the jointing stage with the mixed talcum powder at a 1:200 ratio after raining. HXH was planted every meter in the aisle to serve as an inoculum spreader. The field trials were performed at the using a completely random experimental design. At the booting stage, 20 plants each of common wheat 7182, line CH10A5, the susceptible control HXH, as well as a cluster of *Th. ponticum*, were evaluated three times in the field at the College of Agronomy, Northwest A&F University. Stripe rust resistance was quantified by immunity types (ITs) using a 0–4 scale in which an IT of 0–2 indicated resistance and an IT of 3–4 indicated susceptibility (Wang et al., 2020b).

At the adult stage, powdery mildew resistance trials were performed in the nursery by spontaneous inoculation over three growing seasons (2016–2019). SY225 was planted every meter in the aisle to serve as an induction bed. Twenty plants each of 7182, CH10A5, and the susceptible control SY225, as well as a cluster of *Th. ponticum*, were assessed. Plants were investigated three times for infection symptoms until 20 days after flowering (Zhuang et al., 2015). Powdery mildew resistance was evaluated on a 0–9 scale, which an IT of 0–4 indicated resistance and an IT of 5–9 indicated susceptibility (Sheng and Duan, 1991).

### Molecular Marker Development

SLAF-seq was performed by the Beijing Biomarker Technologies Corporation (Sun et al., 2013). Quality-checked genomic DNA was digested by the restriction enzyme *HaeⅢ*. The Illumina HiSeq platform was used for sequencing after the libraries had passed quality inspection. Dual indexing was used to ensure accurate demultiplexing of the sequence reads for each sample. The data filtering steps were as follows: (1) reads with adapter sequence were removed, (2) reads with an N content exceeding 10% were removed, and (3) reads in which more than 50% of the bases had quality scores less than 10 were removed. Sequence quality and data volume were evaluated after filtering the sequence reads (Chen et al., 2013).

To obtain sequences specific to 1J^s^, sequences with 0% similarity to CS (IWGSC-RefSeq-v1.0) were selected using the Burrows-Wheeler Alignment (BWA) tool, and from these, sequences with at least 95% similarity to *Th. ponticum* were selected. Primers for these sequences were developed using Primer3Plus (http://www.primer3plus.com/). After PCR, specific bands were detected on a 1% agarose gel (Liu et al., 2018b). A Venn diagram and an Upset plot were constructed using TBtools (http://mdr.tbtools.org/).

## Results

### Stability Evaluation of CH10A5

**Figure 1:**
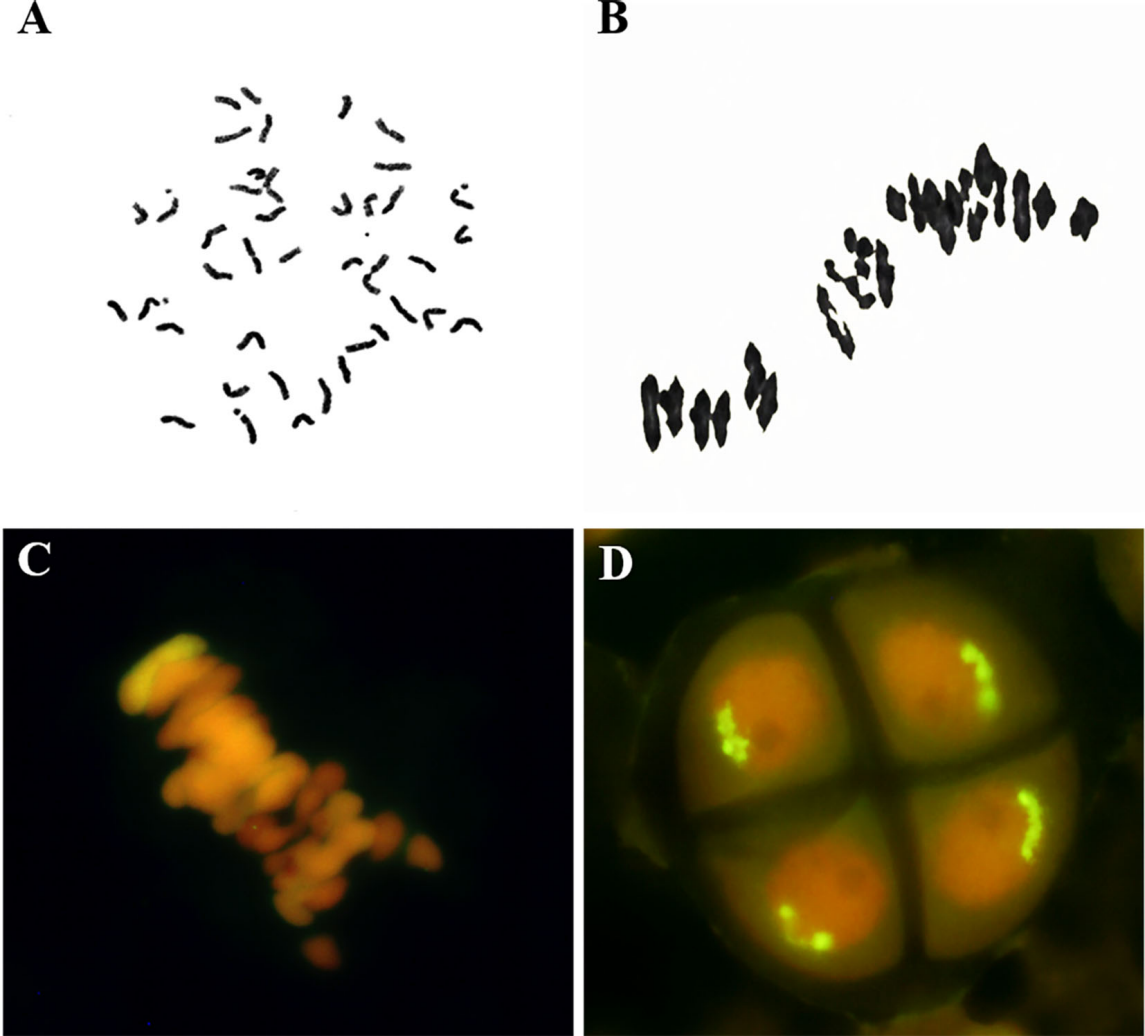
Cytological observations of CH10A5. **(A)** Mitosis stage of root tip cells, 2*n* = 42. **(B)** Meiosis stage of pollen mother cells, 2*n* = 21II. **(C, D)** GISH detection of CH10A5. The yellow-green fluorescent signals identify *Th. ponticum* chromosomes in meiosis metaphase. Chromosomes were counterstained with propidium iodide (red).

The CH10A5 line was derived from the F_7_ progeny of a 7182/*Th. ponticum*//7182 cross. The morphology of CH10A5 was similar to that of common wheat, with a compact plant shape and full grain (**Supplementary Figure 1**). Somatic cell chromosomes from mitotic root tip cells were counted. Of the 281 root tip cells observed, 277 (98.57%) had 42 chromosomes (**Figure 1A**), three cells (1.07%) had 41 chromosomes, and one (0.36%) had 43 chromosomes. A total of 177 PMCs were assessed over a 3-year period, of these 169 PMCs were observed 21 pairs of bivalents in meiotic metaphase I in (**Figure 1B**), up to 95.48%, and univalents were observed in 8 PMCs (4.52%), and on trivalents and quadrivalents were observed. The chromosomes were equally separated in a 21 + 21 manner at anaphase of meiosis II. A bivalent with a strong yellow-green *Th. ponticum* hybridization signal was detected by GISH in meiotic metaphase I (**Figure 1C**), and the alien chromosome separated equally in anaphase meiosis II (**Figure 1D**). Cytological observations therefore indicated that CH10A5 was highly stable.

### Chromosome Composition of CH10A5

**Figure 2:**
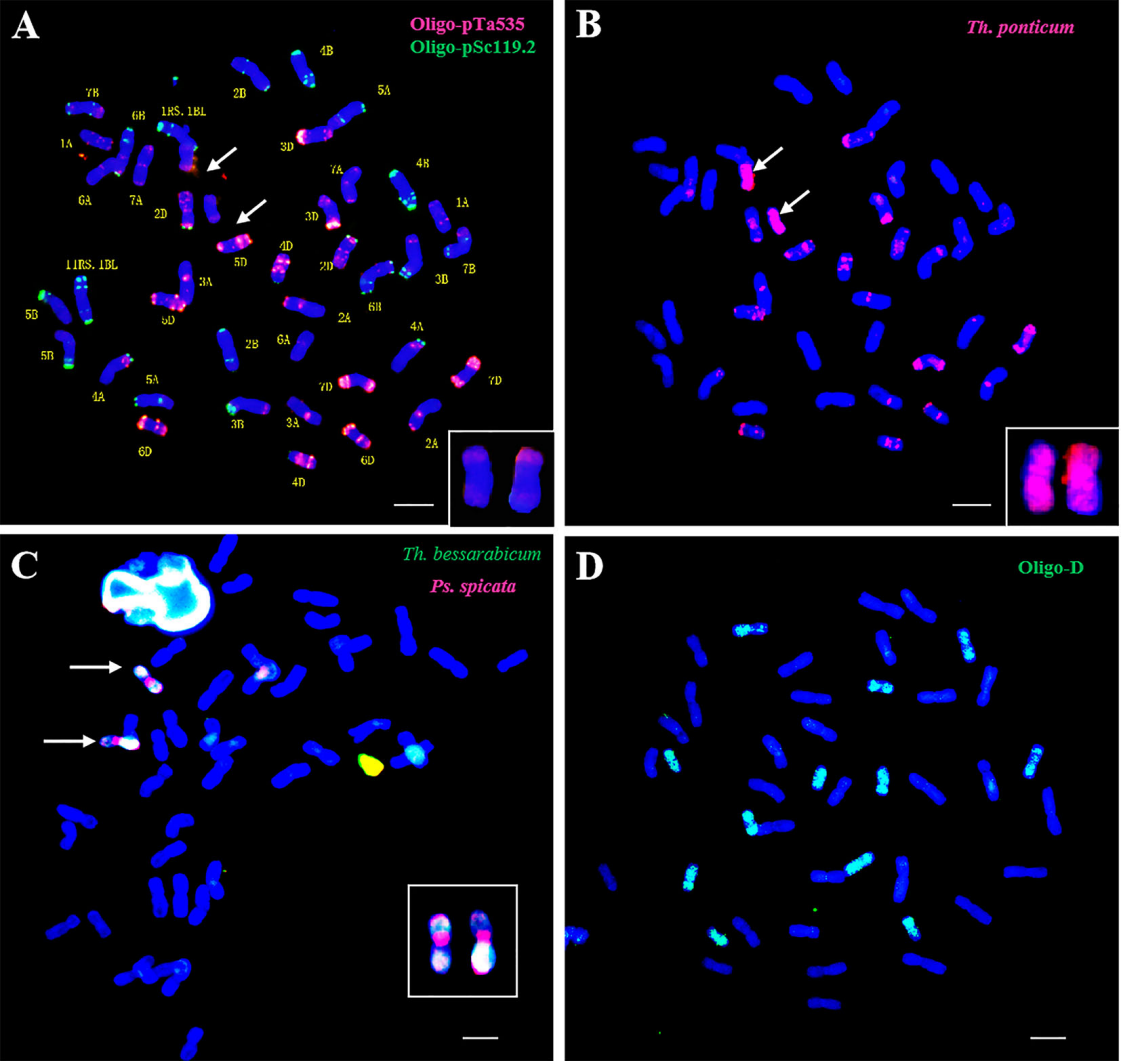
Cytogenetic analysis of CH10A5 by *in situ* hybridization. **(A)** mc-FISH, Oligo-pTa53 5 (red), and Oligo-pSc119.2 (green) were used to perform FISH analysis. **(B)** FISH-GISH, *Th. ponticum* genomic DNA labeled with Texas Red-5-dUTP as a probe and CS genomic DNA as a blocker. The arrows indicate the alien chromosomes in CH10A5. **(C)** mc-GISH, *Th. bessarabicum* genomic DNA (green) and *Ps.spicata* genomic DNA (red) were used as probes. **(D)** ND-FISH, Oligo-D (green) was used to detect 12 D chromosomes. The arrows referred to alien chromosomes. Chromosomes were counterstained with DAPI (blue). Scale bar = 10 μm.

To investigate the chromosome composition of CH10A5, the oligonucleotide probes Oligo-pTa535 (red) and Oligo-pSc119.2 (green) were used for FISH analysis of the CH10A5 chromosomes (**Figure 2A**). Similar to the report of Tang et al. (2014), 40 chromosomes were distinguished in CH10A5, 34 of which had the same FISH signal as CS. A pair of 1RS.1BL translocation chromosomes were observed (Jiang et al., 2017), and signal variation occurred in chromosomes 5A and 6B, and a pair of chromosomes with two arms that resembled the red Oligo-pTa535 signal were not common wheat chromosomes.

Sequential FISH-GISH analysis suggested that the two chromosomes labeled with a bright red signal came from *Th. ponticum* (**Figure 2B**). In addition, mc-GISH analysis showed that two chromosomes had the red signal of *Ps. spicata* in the centromere region and the green signal of *Th. bessarabicum* on both arms. This result indicated that the alien chromosomes belonged to the J^s^ genome (**Figure 2C**). Twelve D-genome chromosomes, rather than the 14 typical of common wheat, were detected in CH10A5 using Oligo-D (**Figure 2D**). Together, these results indicated that the CH10A5 genome was composed of 38 common wheat chromosomes (14A+12B+12D), a pair of 1RS.1BL translocation chromosomes, and two J^s^ chromosomes from *Th. ponticum*.

**Figure 3:**
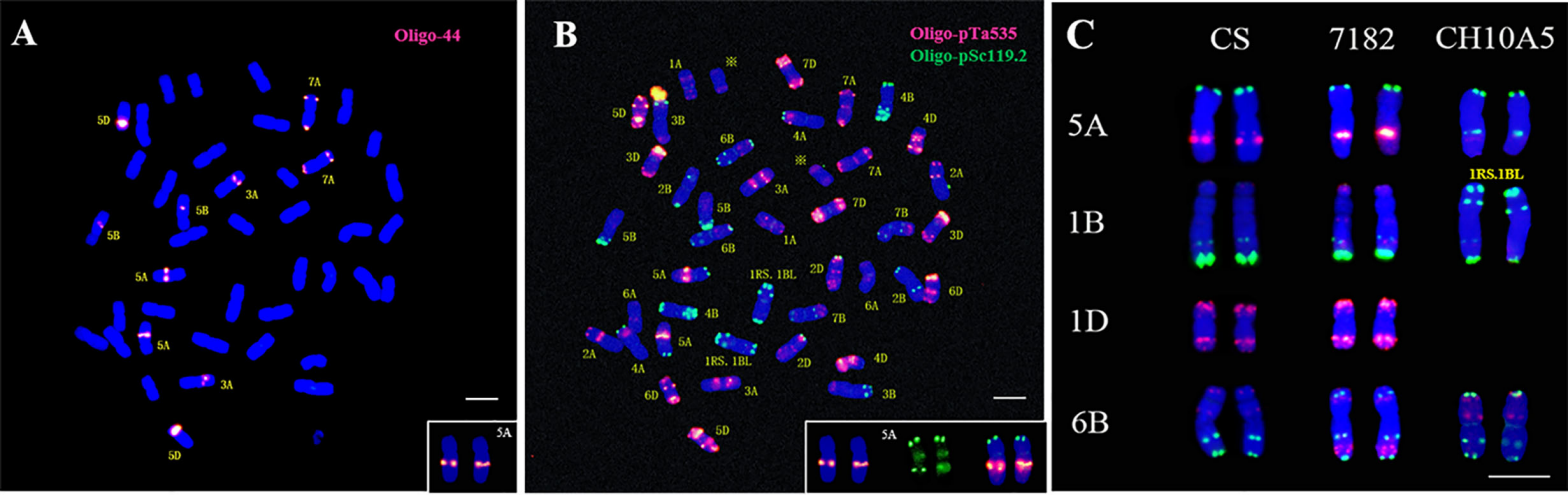
Chromosome variation of CH10A5. **(A)** ND-FISH, Oligo-44 was used to detected 3A, 5A, 5B and 7A; **(B)** mc-FISH, Oligo-pTa535(red), and Oligo-pSc119.2 (green) were used to attest FISH signal variation in 5A with same slides; **(C)** FISH signal comparison between CS, 7182, and CH10A5 on chromosomes 1B, 6B, 5A, and 1D; Chromosomes were counterstained with DAPI (blue). The “※” referred to alien chromosomes, scale bar = 10 μm.

To further characterize the chromosome variations of CH105A, the oligonucleotide probe Oligo-44 were used for ND-FISH analysis. Oligo-44 hybridizes to chromosomes 3A, 5A, 5B, 5D, and 7A, and it was therefore used to demonstrate the variation in 5A signals (**Figure 3A**). A sequential FISH karyotype of CH10A5 was then performed using Oligo-pTa535 and Oligo-pSc119.2 (**Figure 3B**). The results confirmed that the red signal on the long arm of chromosomes 5A in common wheat was changed to a green signal in CH10A5 (**Figure 3C**). Alien chromosomes of *Th. ponticum* had replaced chromosome 1D of common wheat 7182, which may cause FISH signal mutations in chromosomes 5A and 6B.

### Homoeologous Group of the Alien Chromosome

**Figure 4:**
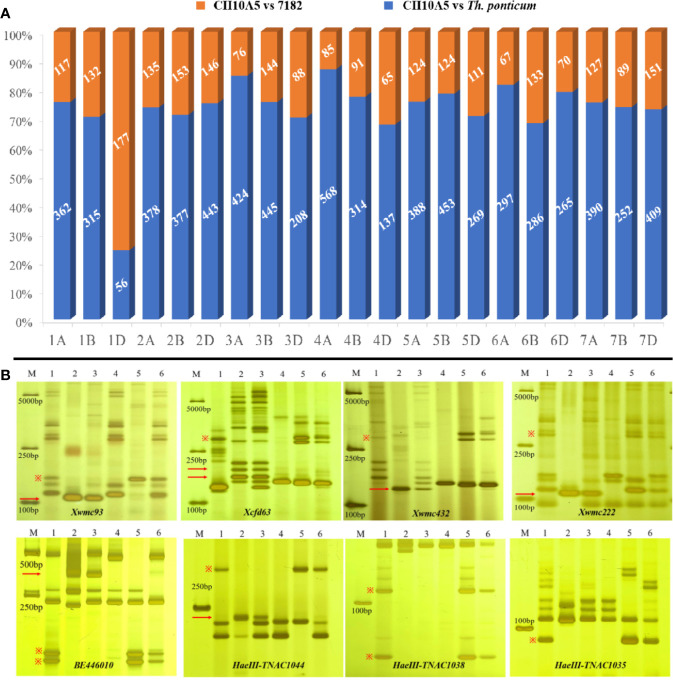
Molecular marker analysis of CH10A5. **(A)** Wheat 15K array analysis. The number refers to the same markers in CH10A5 vs. 7182 (orange) and CH10A5 vs. *Th. ponticum* (blue). **(B)** EST and PLUG marker analysis. M: DL2000 marker; 1–3 refer to 7182, *Th. ponticum*, and CH10A5; 4–6 refer to the CS nulli-tetrasomic materials CSN1DT1B, CSN1BT1D, and CSN1AT1B. The red arrow and “※” symbol represent the distinctive bands of *Th. ponticum* and chromosome 1D, respectively.

Based on wheat array mapping analysis, a total of 12,726, 13,078, and 5,916 polymorphic SNP loci were identified in CH10A5, 7182, and *Th. ponticum*, respectively. A total of 7,258 SNPs were polymorphic between *Th. ponticum* and 7182. The maximum, minimum, and mean percentages of SNP genotyping loci shared between CH10A5 and 7182 were 74.20% (on chromosome 2D), 16.62% (on 1D), and 53.27% overall. The maximum, minimum, and mean percentages of SNP genotyping loci shared between CH10A5 and *Th. ponticum* were 52.52% (on 1D), 11.10% (on 4A), and 19.41% overall (**Supplementary Table 2**). This result confirmed that a large number of wheat-origin SNPs on chromosome 1D were missing from CH10A5 and that CH10A5 and *Th. ponticum* shared the largest proportion of SNP loci on chromosome 1D (**Figure 4A**).

Based on SSR, EST and PLUG marker analysis, 16 markers (4 SSR markers, 4 EST markers, and 8 PLUG markers) amplified the same specific bands in CH10A5 and *Th. ponticum* (**Table 1**, **Figure 4B**). In addition, four SSR markers (*Xwmc93*, *Xcfd63*, *Xwmc432*, and *Xwmc222*), one EST marker (*BE446010*), and three PLUG markers (*TNAC1038*, *TNAC1044*, and *TNAC1035*) demonstrated the deletion of chromosome 1D in CH10A5. All of these markers belong to homoeologous group one.

**Table 1:** Wheat SSR, EST-STS, and PLUG markers used to successfully analyze the chromosomal composition of CH10A5 in this study.

No.	Markers	Type	Primer	Location	Tm (°C)	Size (bp)
1	*Xwmc93*	SSR	F: ACAACTTGCTGCAAAGTTGACG R: CCAACTGAGCTGAGCAACGAAT	1A 1D	61	185, 95
2	*Xcfd63*	SSR	F: TCCTGAGGATGTTGAGGACC R: GAGAGAGGCGAAACATGGAC	1DL	60	200, 280
3	*Xwmc432*	SSR	F: ATGACACCAGATCTAGCAC R: AATATTGGCATGATTACACA	1DS	51	270, 145
4	*Xwmc222*	SSR	F: AAAGGTGCGTTCATAGAAAATTAGA R: AGAGGTGTTTGAGACTAATTTGGTA	1D	60	265, 110
5	*BG313767*	EST-STS	F: GAGGCGTTCTTAAGACGGTG R: GGTGTCAAAAACTTCGCCAT	1AL 1BL 1DL	62	1200
6	*BE497584*	EST-STS	F: CTGTTGCCAAGAGCATTGAA R: GTCACAACATCATCAACCGC	1AL 1BL 1DL	58	760
7	*BF474569*	EST-STS	F: TCCTCAAGGACCCCGACTAC R: TGAAGGGTGAGAGAACTCCG	1AL 1BL 1DL	60	1,500
8	*BE446010*	EST-STS	F: GCATTTTGGAGAGAGCATCA R: ATCTTTTCCATCAGCCCCTT	1AL 1BL 1DL	58	320, 120
9	*TNAC1009-TaqI*	CPLUG	F: CGAACGTGACCATCTACATCA R: CATCTGACTTGGTCTTGGCATA	1AS 1BS 1DS	60	260
10	*TNAC1019-TaqI*	PLUG	F: AACGTGTCCACCGTCTACATC R: CCAGTGGTCTCTGATTCATCC	1AS 1BL 1DL	60	80
11	*TNAC1021-TaqI*	PLUG	F: CTCATGCATGCGTTTGTTAAA R: CCAGCTGAAACAAGCATCTTC	1AL 1BL 1DL	60	80
12	*TNAC1088-HaeIII*	PLUG	F: GGAATCCTTCCTTGTTGAAGA R: AACCTCCGAGTGAAACACAA	1AL 1BL 1DL	60	130
13	*TNAC1026-HaeIII*	PLUG	F: GGGATAGAACTCTGGGACTTCA R: AGTGCCAGGGCATAATACAGC	1AL 1BL 1DL	60	230, 220
14	*TNAC1041-TaqI*	PLUG	F: TCACCACCTCTTTCAGTTGCT R: GAGGAACTCGTCGAGGAAGG	1AL 1BL 1DL	60	820
15	*TNAC1057-TaqI*	PLUG	F: GGAAGATGTGATGCCAACTGT R: AAATATGCCGCCAAGTTAATG	1AL 1BL 1DL	60	510
16	*TNAC1035- HaeIII*	PLUG	F: TGCACTGGGATCTAACCTAAA R: TCCAGTGATCATTTGAAGATTCC	1AL 1BL 1DL	60	120, 60
17	*TNAC1038- HaeIII*	PLUG	F: CCACCAGCTTTCCTTACCATA R: ACTGCTCAATCCAACTGGAAA	1AL 1BL 1DL	60	85
18	*TNAC1044-HaeIII*	PLUG	F: TCAGCAAAGTTCCAGAGAAGG R: GAGGAACTCGTCGAGGAAGG	1AL 1BL 1DL	60	310, 240

The molecular marker results showed that the chromosome deleted in CH10A5 was 1D and that the pair of J^s^ chromosomes carried by CH10A5 belonged to homoeologous group one of *Th. ponticum*. CH10A5 was therefore a wheat–*Th. ponticum* 1 J^s^ (1D) disomic substitution line.

### Assessment of Disease Resistance

**Figure 5:**
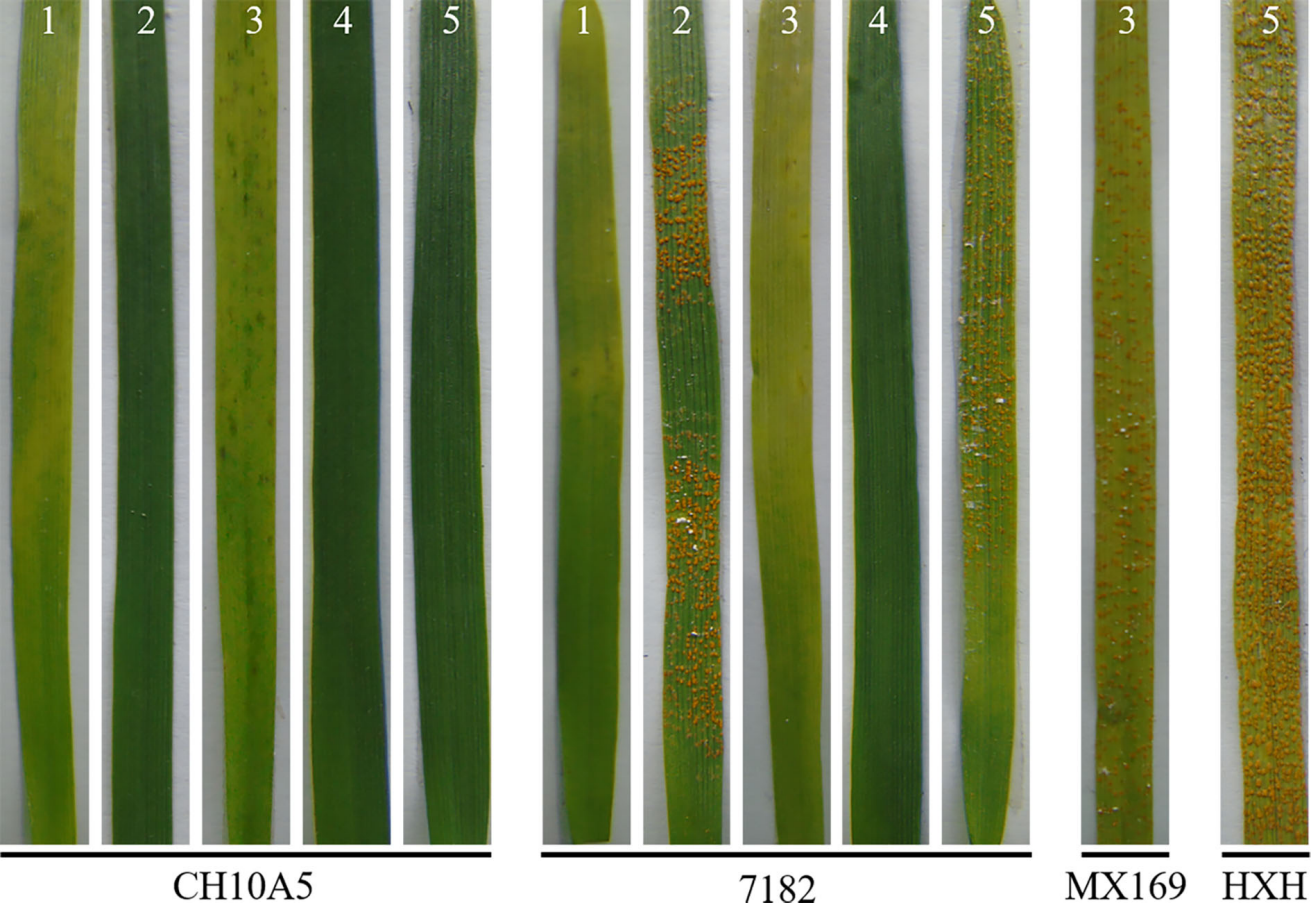
Stripe rust reaction of CH10A5 and 7182 to five *Pst* races at the seedling stage. HXH and MX169 were the susceptible controls. Numbers 1–5 refer to *Pst* races CYR23, CYR29, CYR31, CYR32, and CYR34, respectively.

At the seedling stage, CH10A5 and the common wheat parent 7182 were inoculated with five *Pst* races (CYR23, CYR29, CYR31, CYR32, and CYR34). Ten days after inoculation, the susceptible controls HXH or MX169 had become fully infected. Three successive investigations of disease resistance showed that the immunity types of CH10A5 to CYR29, CYR31, and CYR32 were 0, indicating immunity. The immunity types of CH10A5 to CYR23 and CYR34 were 1, indicating high resistance. By contrast, 7182 was resistant to CYR23, CYR31, and CYR32 and susceptible to CYR29 and CYR34 (**Table 2**, **Figure 5**). The seedling resistance results therefore showed that CH10A5 carried new resistance gene(s) from *Th. ponticum*.

**Table 2:** Stripe rust resistance of CH10A5 and its parents at the adult and seedling stages.

Materials	Year and mixed races at adult stage			Five races at seedling stage				
	2017CYR32 & CYR33	2018CYR31 & CYR32	2019CYR32 & CYR34	CYR23	CYR29	CYR31	CYR32	CYR34
7182	3	3	3	0	4	1	0	4
*Th. ponticum*	0	0	0	–	–	–	–	–
CH10A5	1	0	1	1	0	0	0	1
MX169	–	–	–	4		4	4	–
HXH	4	4	4	4	4	4	4	4

“-” indicates that no data were collected; Mingxian169 (MX169) and Huixianhong (HXH) were used as susceptible controls.

**Figure 6:**
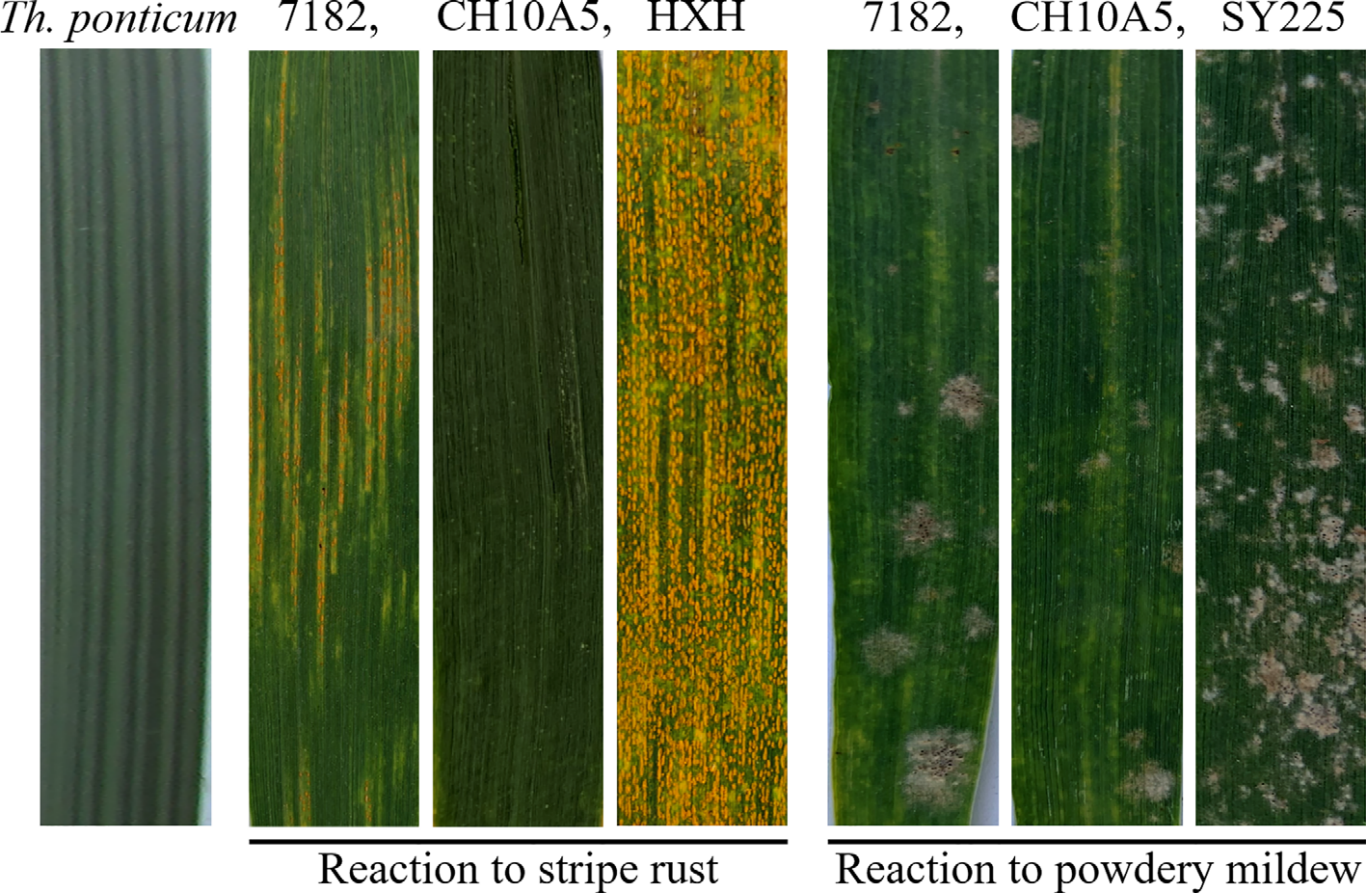
Stripe rust and powdery mildew resistance of CH10A5 and its parents at the adult stage. Huixianhong (HXH) was the susceptible control for *Pst*, and Shanyou225 (SY225) was the susceptible control for *Bgt*.

At the adult stage, resistance to three *Pst* races (CYR31, CYR32, and CYR33) was assessed in CH10A5 and its parents through inoculation experiments performed over three growing seasons (2016–2019). Ten plants were randomly selected to evaluate disease resistance each year. After three consecutive years of investigation, it was found that the stripe rust resistance of each line was consistent. The IT of the susceptible control HXH was 4, indicating high sensitivity. That of CH10A5 was 0 or 1, few small uredia with distinct necrosis on leaves, indicating high resistance; that of *Th. ponticum* was 0, and there were no visible uredia and necrosis on leaves, indicating immunity; and that of 7182 was 3, there were a lot of medium-sized uredia, no necrosis, but with chlorosis on leaves, indicating moderate sensitivity (**Table 2**, **Figure 6**). These results suggested that the chromosome 1J^s^ of CH10A5 might carry on a broad-spectrum resistance gene to stripe rust.

CH10A5 and its parents were also planted in a nursery where powdery mildew was present. When the powdery mildew spores were fully developed on the leaves of the susceptible control SY225, the ITs of the four materials were as follows: SY225, IT = 9, highly susceptible to powdery mildew; 7182, IT = 5, moderate susceptible to powdery mildew; CH10A5, IT = 3, high resistance to powdery mildew; *Th. ponticum*, IT = 0, immune to powdery mildew. Thus, the material with chromosomes from *Th. ponticum* exhibited substantial resistance to powdery mildew (**Table 3**, **Figure 6**). Together, the results of the disease resistance trials indicated that the alien chromosomes of CH10A5 carried gene(s), which probably responsible for resistance to stripe rust and powdery mildew.

**Table 3:** Powdery mildew reaction of CH10A5 and its parents at the adult stage in 2016–2019.

Materials	Year	Mixed races at adult stage		
		No. of observed plants	Infection type	Resistance/susceptibility
*Th. ponticum*	2017 May	cluster	0	R
	2018 May	cluster	0	R
	2019 May	cluster	0	R
7182	2017 May	20	5	MS
	2018 May	20	5	MS
	2019 May	20	5	MS
CH10A5	2017 May	20	3	R
	2018 May	20	3	R
	2019 May	20	3	R
SY225	2017 May	20	9	S
	2018 May	20	9	S
	2019 May	20	9	S

R, MS, and S refer to resistance, moderate susceptibility, and susceptibility, respectively.

### Molecular Marker Development

After SLAF sequencing, a total of 11,931,086, 10,383,011, and 9,871,061 reads were obtained from CH10A5, 7182, and *Th. ponticum*, with an average Q30 score of 90.34% and an average GC content of 46.55%. The final SLAF numbers were 467,788, 157,996, and 500,275, and the average sequencing depth was 17.93. Using the BWA tool, 1220 reads were found to have 0% similarity to the CS wheat reference genome (IWGSC-RefSeq-v1.0). Nine hundred fifty-seven of these reads had at least 95% similarity to *Th. ponticum* and were considered to be specific fragments of chromosome 1J^s^.

To develop *Th. ponticum* 1J^s^ chromosome-specific markers, 957 pairs of STS primers were designed based on these specific fragments and used to amplify sequences from CS, 7182, *Th. ponticum*, and CH10A5. Compared with CS and 7182, 507 STS primers were found to be specific to CH10A5 and *Th. ponticum* through PCR and electrophoresis detection (**Supplementary Figure 2**, **Supplementary Table 3**). The success rate for the development of markers specific to the alien chromosome using SLAF-seq was therefore reached 52.98%. Finally, 507 chromosome sequences of 1J^s^ were obtained (unpublished data). Some chromosome sequences were related to plant disease resistance, which helped us to locate the stripe rust and powdery mildew resistance genes on chromosome 1J^s^.

**Figure 7:**
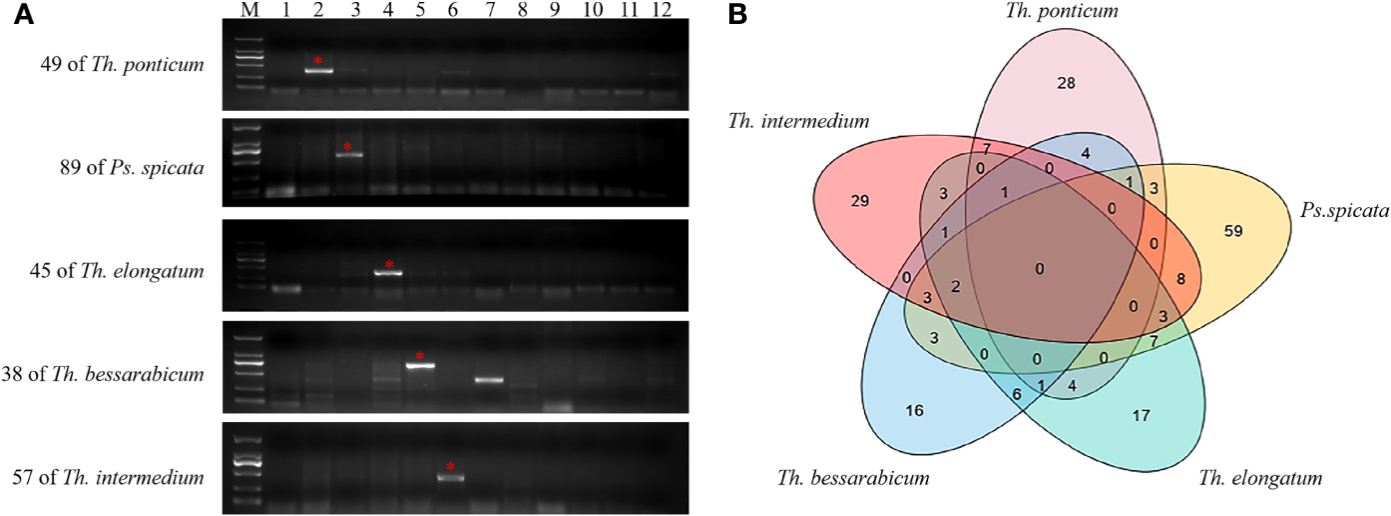
Molecular marker development and PCR amplification in 7182 and other wheat-related species. **(A)** Specific markers for *Th. ponticum*, *Ps. spicata*, *Th elongatum*, *Th. bessarabicum*, and *Th. intermedium*. Lanes: M, DL2000; 1–12 refer to 7182, *Th. ponticum*, *Ps. spicata*, *Th. elongatum*, *Th. bessarabicum*, *Th. intermedium*, *L. mollis*, *Ps. huashanica*, *S. cereale*, *Ae. geniculata*, *T. urartu*, and *Ae. tauschii*, respectively. The red stars and arrows indicate target bands. **(B)** Venn diagrams show the number of specific markers for *Th. ponticum, Th. bessarabicum, Th. elongatum*, *Ps. spicata*, and *Th. intermedium*. Overlaps represent the number of markers that were shared among two or three species.

To evaluate the polymorphisms and utility of the chromosome 1J^s^-specific markers, PCR was performed on 7182 and eleven wheat-related species to identify markers that amplified specific bands that distinguished 7182 from other wheat-related species. The numbers of specific markers for each species were as follows: 49 for *Th. ponticum*, 38 for *Th. bessarabicum*, 45 for *Th. elongatum*, 89 for *Ps. spicata*, and 57 for *Th. intermedium* (**Figure 7A**). In addition, Venn diagrams showed that some markers were shared by two or three species (**Figure 7B**). PCR of other wheat-related species also amplified specific bands. There were 70, 51, 67, 54, 17, and 43 markers for *L. mollis*, *Ps. huashanica*, *S. cereale*, *Ae. geniculata*, *T. urartu*, and *Ae. tauschii*, respectively (**Supplementary Table 4**).

## Discussion

Since the 1960s, at least 12 genes for resistance to fungal or viral diseases have been reported from *Th. ponticum* in the form of chromosome translocations. Among them, two leaf rust resistance genes (*Lr19* and *Lr29*), two stem rust resistance genes (*Sr25* and *Sr43*), and one FHB gene (*Fhb7*) were located on homoeologous seven; *Sr24* and *Lr24* were located on homoeologous three; and *Sr26* and *SrB* were located on homoeologous six (Li and Wang, 2009; Niu et al., 2014; Guo et al., 2015; Kuzmanovic et al., 2019; Mago et al., 2019). A wheat–*Th. ponticum* introgression line was resistant to powdery mildew (*Pm51*) and stripe rust (*Yr69*), and the resistance genes were all located on homoeologous two (Zhan et al., 2014; Hou et al., 2016). Likewise, resistance to eyespot and stripe rust at the adult stage was associated with homoeologous groups four and five in wheat–*Th. ponticum* line (Li et al., 2004; Mo et al., 2017). The cloning of *Fhb7* will greatly promote the development of wheat FHB resistance breeding in China (Wang et al., 2020a). However, stable addition, substitution, and translocation wheat–*Th. ponticum* lines are rarely reported, particularly for homoeologous group one, and it is therefore important to explore potential resistance genes in *Th. ponticum*. In this study, the wheat–*Th. ponticum* 1J^s^ (1D) disomic substitution line CH10A5 was probably resistant to both stripe rust and powdery mildew. This is first report of stripe rust and powdery mildew resistance probably associated with chromosome 1J^s^. By comparing the resistance response of CH10A5 to that of 7182, we demonstrated that chromosomes 1J^s^ of *Th. ponticum* might possess new resistance gene(s) for stripe rust and powdery mildew. CH10A5 is therefore an important material for the exploration of potential resistance genes from *Th. ponticum*. For further use of this 1J^s^ (1D) disomic substitution line, small fragment translocation lines will be developed using radiation and the *Ph1b* gene (Zheng et al., 2006; Dai et al., 2020) to explore the segments of resistance genes and identify new useful traits.

FISH analysis can easily and efficiently detect different structural rearrangements and polymorphic blocks in wheat chromosomes (Huang et al., 2018). The introduction of an alien chromosome may lead to structural changes or altered gene expression of common wheat chromosomes (Rey et al., 2018). Previous studies have indicated that structural alterations of wheat chromosomes occur randomly (Yang et al., 2016), but Huang et al. (2018) have suggested that chromosomes 1B, 1D, 4A, and 5B undergo more rearrangements. Previous studies have shown that different wheat*–Th. ponticum* addition or substitution lines exhibit random chromosomal variations (Chen et al., 2012; Zheng et al., 2014; Pei et al., 2018; Wang et al., 2019). The 1RS.1BL translocation was successfully identified by using Oligo-pSc119.2 (6-FAM-5’) and Oligo-pTa535 (Tamra-5’) (Jiang et al., 2017), and a pair of 1RS.1BL translocation chromosomes was detected in CH10A5, which may occurred the cross pollination of wheat during breeding process. In this study, the FISH signals of chromosomes 5A and 6B were changed in CH10A5 (see **Figure 3**), but no change was observed in the FISH signals of the D genome, indicating that chromosome J^s^ caused chromosomal rearrangement of the A and B genomes. This result confirms that the St or E genome is more closely related to the D genome than to the A and B genomes, consistent with previous studies (Liu et al., 2007). The researchers have constructed a FISH-based karyotype of wheat relatives using oligonucleotide probes, such as *Th. bessarabicum*, *Th. elongatum* (Grewal et al., 2018; Li et al., 2018), and *Th. intermedium* (Cui et al., 2018). We concluded that the chromosome 1E of *Th. elongatum* could be hybridized with strong signal using Oligo-pSc119.2 (green) and Oligo-pTa535 (red). Chromosomes 1J of *Th. bessarabicum* had Oligo-pSc119.2 signals (green) on terminal regions of both arms, and the FISH pattern of chromosome *Th. intermedium* was not very clear, but the GISH pattern was different from the chromosomes 1J^s^ in CH10A5. In our study, the FISH pattern of 1J^s^ was hybridized with weak signal using Oligo- pTa535 (red), but not introducted the signal of Oligo-pSc119.2. In conclusion, the results suggested that chromosomes 1J^s^ in CH10A5 derive from *Th. ponticum*.

Molecular markers play an important role in tracking alien genetic material in a wheat background and are widely used in genetic breeding, genomic mapping (Zhou et al., 2018), gene localization (Zhang et al., 2018), identification of species relatedness (Wang et al., 2017; Cseh et al., 2019), gene bank construction (Zhang et al., 2002), gene cloning, and other applications (Ma and Tomita, 2013). The lack of rich and reliable molecular markers and the shortage of high-density linkage maps are significant constraints for research work. Some specific molecular markers have been obtained from wheat-related species by SLAF-seq (Chen et al., 2013), and SLAF-seq technology is widely used in molecular marker development for wheat and related species. Chen et al. (2013) developed a large number of *Th. elongatum* 7E chromosome-specific molecular markers. Liu et al. (2018a) constructed a physical map of chromosome 4Ag from *Th. ponticum* and obtained 89 blue-grain-related molecular markers; 67 markers specific to *Th. ponticum* were developed in a separate study (Liu et al., 2018b). All of these markers were developed using SLAF-seq. In this study, some markers were associated with resistance to disease, making them using for the identification and study of related genes. Here, specific markers for *Th. ponticum* (49 markers), *Th. bessarabicum* (38), *Th. elongatum* (45), *Ps. spicata* (89), and *Th. intermedium* (57) were also obtained using SLAF-seq, and a number of markers had polymorphisms in two or all five species. These results indicate that chromosome 1J^s^ in CH10A5 may undergo chromosome rearrangements between the J and St genomes, consistent with previous studie (Kruppa and Molnar-Lang, 2016; He et al., 2017). These molecular markers could help us further study the relationships among wheat-related species and detect the introgression of foreign genes.

## Conclusions

CH10A5 is a novel 1J^s^ (1D) disomic substitution line derived from a cross between common wheat 7182 and *Th. ponticum*. The chromosome composition of CH10A5 is 14A+12B+12D+1RS.1BL+1J^s^. Moreover, it possesses resistance gene(s) to both wheat stripe rust and powdery mildew at the adult stage. In addition, 507 chromosome-specific markers for 1J^s^ were obtained based on SLAF-seq, of which 49 were helpful for characterizing the genetic material of *Th. ponticum*. All these specific markers will be useful for revealing the genetic diversity among wheat-related species and accelerating the process of molecular marker-assisted breeding. The novel disomic substitution line CH10A5 can be exploited as a promising germplasm for wheat resistance breeding and chromosome engineering.

## Data Availability Statement

The datasets for this article are not publicly available because: a large number of verification experiments are still under way. Requests to access the datasets should be directed to WJ, jiwanquan2008@126.com.

## Author Contributions

WJ, CW, and HZ designed the research. YanW, QC, JZ, and SW performed the research. CC and CW contributed to the development of the material. YanW, CQ, and JZ collected the samples for DNA extraction and performed the genotyping. YanW analyzed the data. HZ, CW, and YajW contributed to the writing of the article.

## Funding

This work was supported by the National Key Research and Development Program of China (No.2016YFD0102000).

## Conflict of Interest

The authors declare that the research was conducted in the absence of any commercial or financial relationships that could be construed as a potential conflict of interest.

## Supplementary Material

Supplementary Figure1 Agronomic performance of CH10A5 and its parents. Letters **(A**–**C)** refer to plant, spike, and spikelets; numbers 1, 2, and 3 stands for CH10A5, 7182, and *Th. ponticum*, respectively.

Supplementary Figure 2 Molecular marker development of CH10A5. Lanes 1, 2, 3 and 4 correspond to CS, 7182, *Th. ponticum*, and CH10A5, respectively. “*” represents the specific bands.

Supplementary Table 1 All 72 wheat EST-STS and 135 of PLUG markers used to analyze the chromosomal composition of CH10A5.

Supplementary Table 2 Genotype data from wheat 15K SNP arrays for CH10A5 and its parents.

Supplementary Table 3 507 specific primers for *Th. ponticum*.

Supplementary Table 4 The number of specific markers for each wheat relative species.
