# Supplementary figures and images for: Cytogenetic Analysis and Molecular Marker Development for a New Wheat–Thinopyrum ponticum 1Js (1D) Disomic Substitution Line With Resistance to Stripe Rust and Powdery Mildew
Source: Front Plant Sci. 2020 Aug 21;11:1282. doi: 10.3389/fpls.2020.01282 (PMC7472378; doi:10.3389/fpls.2020.01282)

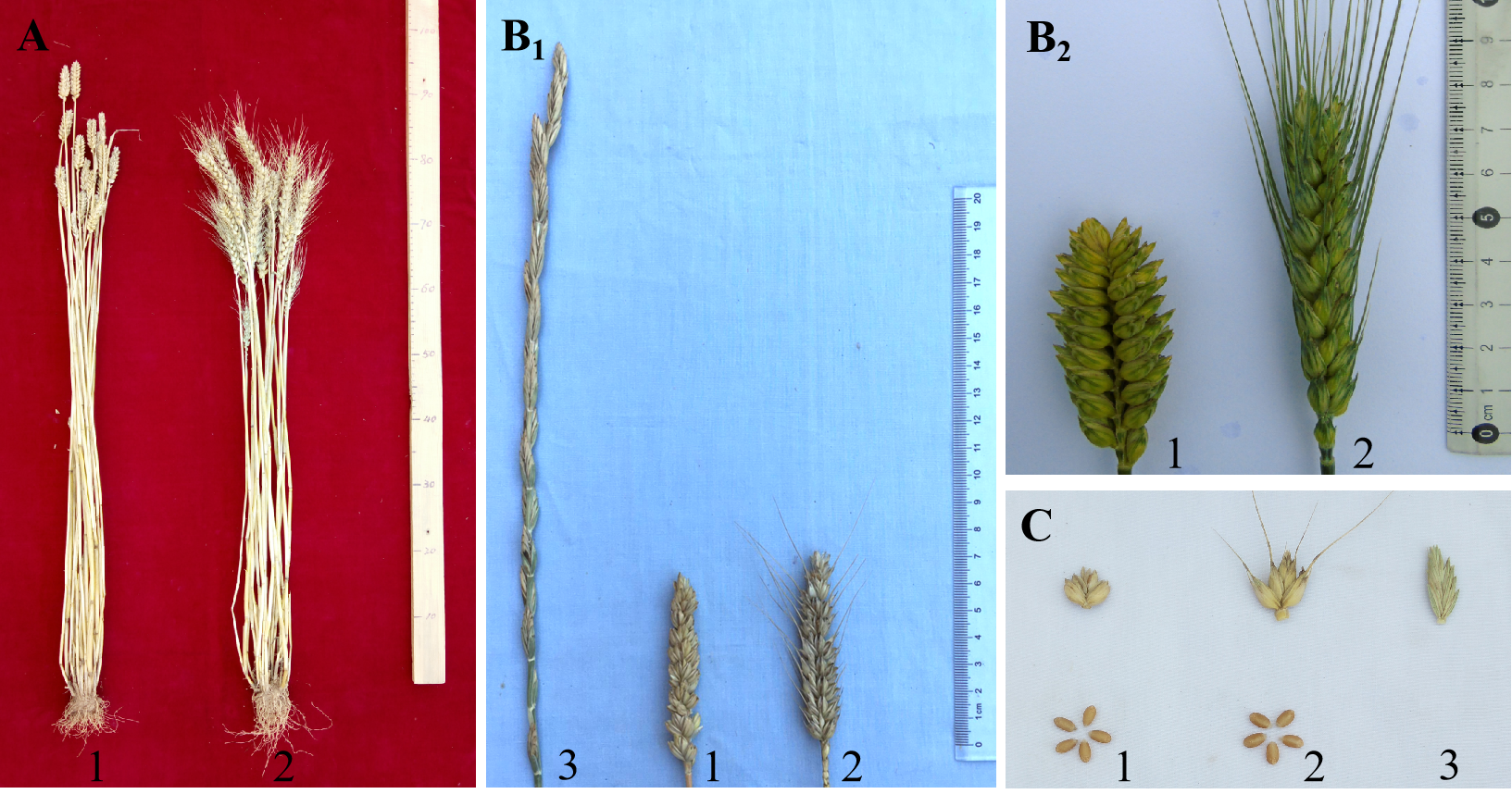

Supplement: Supplementary Figure1 — Agronomic performance of CH10A5 and its parents. Letters (A–C) refer to plant, spike, and spikelets; numbers 1, 2, and 3 stands for CH10A5, 7182, and Th. ponticum, respectively. [file Image_1.tif]

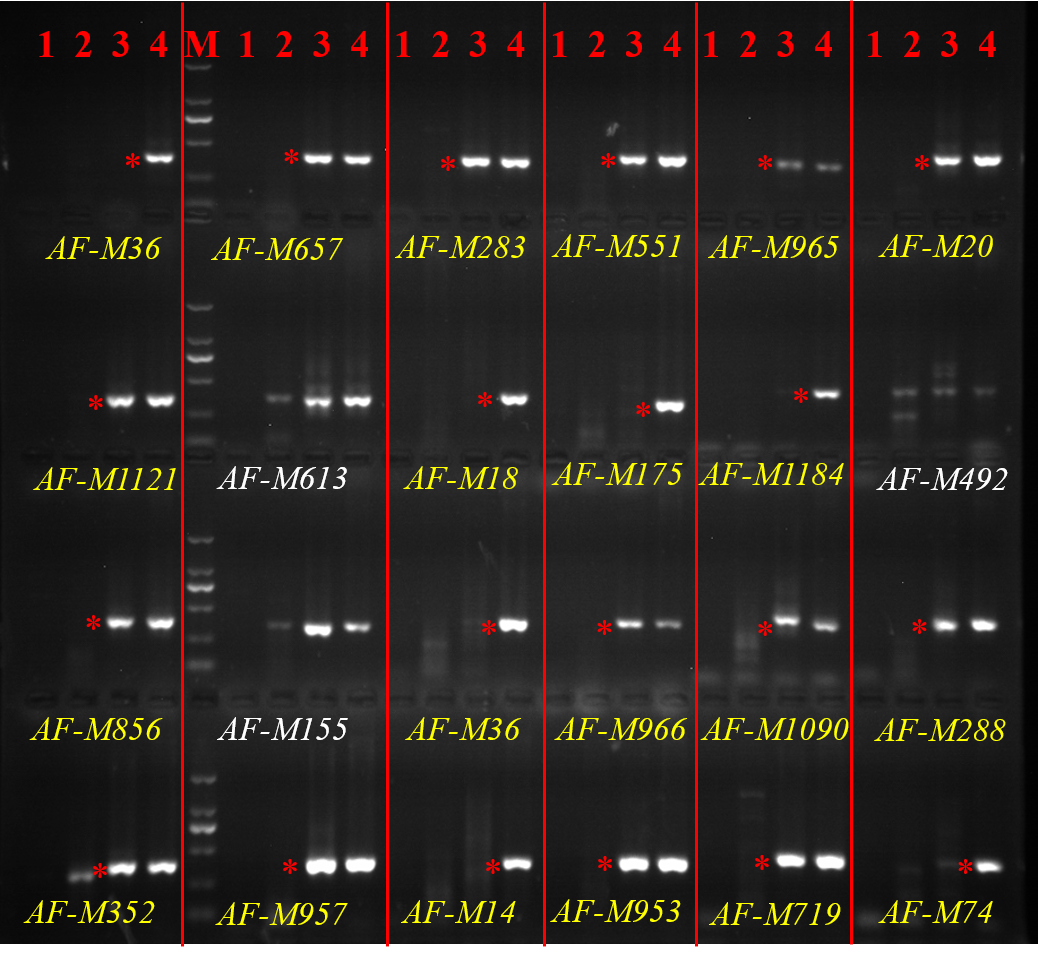

Supplement: Supplementary Figure 2 — Molecular marker development of CH10A5. Lanes 1, 2, 3 and 4 correspond to CS, 7182, Th. ponticum, and CH10A5, respectively. “*” represents the specific bands. [file Image_2.tif]
